# The use of computer-aided analysis of facial expressions based on the support vector machine in the diagnosis of ASD

**DOI:** 10.1192/j.eurpsy.2025.654

**Published:** 2025-08-26

**Authors:** K. Kania, K. Wilczyński, D. Pęszor, L. Cichoń, K. Wojciechowski, A. Kawalec, M. Janas-Kozik

**Affiliations:** 1 Medical University of Silesia, Katowice; 2 Department of Psychiatry and Psychotherapy of Developmental Age, Medical University of Silesia, Sosnowiec; 3 Research and development center, Polish-Japanese Academy of Information Technology. Warsaw, Poland

## Abstract

**Introduction:**

Autism spectrum disorder (ASD) is characterized by communication challenges, particularly in non-verbal aspects such as facial expressions.Research in this area is limited due to the lack of accurate methodologies. Existing literature generally agrees that individuals with ASD often show a disconnect between verbal communication and emotional expression, with facial expressions being diminished or inappropriate. Most studies have relied on ratings by highly trained observers, which can reduce accuracy and introduce biases, such as confirmation bias.

**Objectives:**

Our goal was to create a model for capturing and analyzing facial expressions using computer algorithms and assess its effectiveness in identifying individuals with ASD.

**Methods:**

The study involved 100 participants, divided into two groups based on ASD diagnosis. The ASD group included 73 individuals, 51 (69.8%) of whom were male, while the control group comprised 27 participants, with 16 (59.2%) being male. ASD diagnoses were made by a specialist child and adolescent psychiatrist using developmental history and mental state examinations, confirmed with the ADOS-2 protocol. In the control group, ASD was ruled out using the same protocol. A significant age difference was found between the ASD group (mean age: 14 years; 95% CI: 13.5-14.5) and the control group (mean age: 16.3 years; 95% CI: 15.2-17.5), according to the Mann-Whitney U test.

All participants completed three tasks: a semi-structured conversation, recognizing facial expressions displayed on a screen, and imitating these expressions. Throughout the tasks, participants’ faces were recorded using five cameras positioned around them. Faces were then detected in the images using a sliding window algorithm in a multi-resolution representation of the Gaussian pyramid utilizing a linear classifier based on the Support Vector Machine (SVM) with a classical Histogram of Oriented Gradients (HOG) descriptor. An Ensemble of Regression Trees was applied to these detected faces to model facial landmarks in each frame. Using these landmarks, anthropometric distances and proportions were calculated, which were then used to train the SVM classifier.

**Results:**

The obtained model was able to predict the diagnosis of ASD in the study population with almost 100% accuracy. The mean difference between the probability of the correct class and the probability of the incorrect class determined by the SVM on the test set was 56%.

**Conclusions:**

This method of facial expression analysis using an SVM classifier shows potential as a tool for diagnosing ASD. The technique could be applied using smartphones. However, further research is needed to evaluate its clinical viability, particularly when using non-standard devices. These findings also support the hypothesis that individuals with ASD display facial expressions significantly differently from neurotypical individuals.

**Disclosure of Interest:**

None Declared

